# Molecular Serum Albumin Unmask Nanobio Properties of Molecular Graphenes in Shungite Carbon Nanoparticles

**DOI:** 10.3390/ijms25052465

**Published:** 2024-02-20

**Authors:** Sergey Rozhkov, Andrey Goryunov, Natalia Rozhkova

**Affiliations:** 1 Institute of Biology, Karelian Research Centre RAS, 185910 Petrozavodsk, Russia; rozhkov@krc.karelia.ru (S.R.); goryunov@krc.karelia.ru (A.G.); 2 Institute of Geology, Karelian Research Centre RAS, 185910 Petrozavodsk, Russia

**Keywords:** serum albumin, shungite carbon nanoparticles, fatty acids, redox bioactivity, protein corona, metastable clusters

## Abstract

Serum albumin is a popular macromolecule for studying the effect of proteins on the colloidal stability of nanoparticle (NP) dispersions, as well as the protein–nanoparticle interaction and protein corona formation. In this work, we analyze the specific conformation-dependent phase, redox, and fatty acid delivery properties of bovine albumin in the presence of shungite carbon (ShC) molecular graphenes stabilized in aqueous dispersions in the form of NPs in order to reveal the features of NP bioactivity. The formation of NP complexes with proteins (protein corona around NP) affects the transport properties of albumin for the delivery of fatty acids. Being acceptors of electrons and ligands, ShC NPs are capable of exhibiting both their own biological activity and significantly affecting conformational and phase transformations in protein systems.

## 1. Introduction

### 1.1. Serum Albumin and Shungite Carbon Nanoparticles

In recent years, nanoparticles have been widely used in practical biomedicine [1]. This is due to their high penetrating ability into tissues and cells, the ability to transport biologically active substances, their redox properties, and their effects on the structural organization of biosystems [2]. Simultaneously, more and more data are accumulating on the potential risk of the long-term use of nanoparticles [3] and their toxicity to the environment [4]. Inorganic nanoparticles based on silicon oxide, various metals, their oxides, and sulfides, as well as quantum dots and carbon nanoparticles, attract special attention. However, insufficient knowledge of the possible solvent-dependent physicochemical mechanisms of the interaction of nanoparticles with biological objects and with protein systems, in particular, leads to the absence of theoretical models that make it possible to predict the result of nanoparticle interactions with biosystems [5]. 

Carbon nanomaterials present the best form of abiogenic nanomaterials found in the environment. In particular, natural shungite carbon (ShC) is widely used in cosmetics and foods without even passing full safety tests [6]. It is actively used for various cosmetic and balneological purposes, as bioadditives, and filters for water treatment. However, such an application has a purely marketing value without sufficient scientific justification. In spite of a firmly justified molecule-based structure of the solid [7], the molecular mechanisms of its biological activity have not been characterized enough. At the same time, the development of green technologies [8] for obtaining relatively large amounts of ShC nanoparticles (ShC NPs), which are characterized by structural homogeneity [9], makes ShC a promising material for bionanomedicine purposes. 

Shungites are widespread in Karelia (Russia) as rocks of the Precambrian age [10,11]. They generally present a mixture of nanosized crystalline silica with *sp*^2^ carbon, the mass content of which varies from 5% to 98%, depending on the deposit. Shungite rocks originated in soft geological conditions (temperature less than 450 °C, pressure 70 MPa), and the water environment played a key role in their formation process. ShC can be extracted from carbon-rich shungite rocks based on green chemistry methods [10] with the formation of stable dispersions of nanoparticles (ShC NPs) in the aquatic environment. A possible scenario for the origin of the ShC was proposed [12] based on the hydrothermal impact in the condition of an oxygen anomaly on the graphite layers that existed in the region in the Precambrian period. Subsequently, oxidized graphenes were subjected to reduction processes over 1.8 billion years in a water environment. At the same time, the presence of residual and constitutional water in the nanocarbon structure makes it easy to release ShC NPs into the aquatic environment, thereby ensuring the unique properties of ShC to re-disperse when water is added.

ShC NPs are formed by basic structural units (BSUs) less than 1 nm, which are necklaced graphene molecules. This reflects the idea that graphene molecules are always terminated by heteroatoms, which form a kind of “necklace” on the graphene domains [7]. It was shown empirically and virtually that the BSUs belong to a large family of reduced graphene oxides (rGOs). In a liquid environment, they retain their characteristic tendency to form nanosized stacks, thus self-associating and forming nanoparticles [10,12]. The morphology and spectral properties of the obtained ShC dispersions depend on the liquid in use. The most stable dispersions with NP concentrations of ca 0.1 mg/mL are formed in water, although the dispersions in toluene and other solvents [13] can be produced as well. 

Serum albumin (SA) has a broad perspective in the creation of bionanoconjugates with nanoparticles for the regulation of the protein assembly into fibrils and the disaggregation of preformed fibrils [14], as well as being an ideal candidate for nanoparticle formulation as potential controlled-release drug delivery systems [15]. In addition, SA has been used for surface coating and passivation in biological liquids [16,17] and as biosurfactants [18]. In turn, SA is used as a sensor of conformational changes when interacting with surfaces of various natures [19]. Such a range of possible applications of albumin is due to both the availability and cheapness of SAs and the relative completeness of knowledge [16,20,21] of their conformational state, intermolecular interaction, colloidal stability [22], and phase behavior in dispersions with different microenvironments. 

**Figure 1:**
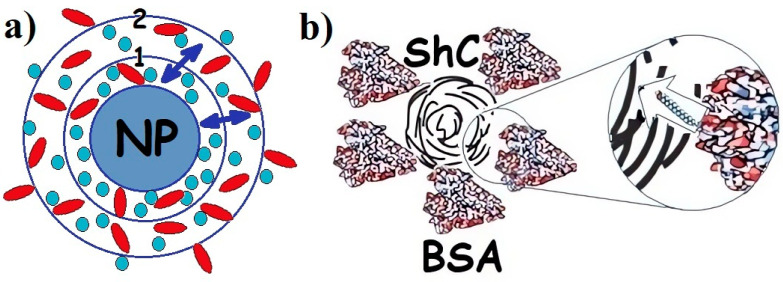
(**a**) Schematic presentation of protein corona in the dispersion of any nanoparticle (NPs) with proteins of different types (adapted from [1]): 1—hard corona; 2—soft corona; (↔)—exchange of proteins between hard and soft corona. Proteins are divided between hard and soft corona depending on the affinity of the proteins for the NP surface. (**b**). Corona of bovine serum albumin (BSA) around shungite carbon NP. Insert: long rod indicates exchange of fatty acids (which are not a part of the corona) between BSA and NP. Different colors are provided for ease of observation.

When the biological environment interacts with the surface, colloidal nanomaterials, nanoparticles, a dynamic coating of biomolecules, and proteins spontaneously arise around them, called the protein corona [19,23,24] (Figure 1). Irreversible or at least long-term binding of a protein by a nanoparticle leads to the formation of a “hard corona”, whereas the rapidly reversible binding of a protein that has a higher turnover rate results in the formation of a “soft corona”. The prospects for the wide use of nanoparticles in biomedicine [1,3] suggest the need to study this phenomenon since the toxicity of nanomaterials and the nanorisks of their use largely depend on the functioning of the corona. Blood plasma proteins, and in particular albumins, are the most important components in the formation of the protein corona, although the mechanism of its formation is complex and insufficiently studied [25], being a phase-regulating process.

Highly organized protein molecules are edited by evolution to perform specific functions. However, other supramolecular structures and nanoparticles have been intensively introduced into living systems for possible adjustment of these functions. It is natural to assume that, in this case, the introduced structures, to some extent, must also fulfill a certain norm of function per unit of structure, characteristic of biomacromolecules. However, research in this direction performed in this work is only at the initial stage. Albumin is interesting for its specific structure–function features discussed in Section 1.2, which make it possible to study the effect of nanoparticles on the most important physiological functions of SA associated with transport, phase, colloidal, redox, and conformational properties. In this regard, the question arises whether the nanoparticles themselves are capable of performing similar functions characteristic of albumin. It can be assumed that in the case of such abilities, hybrid dispersions of albumin and nanoparticles will be the most productive in biomedical applications due to the possibility of mutually complementing and regulating functions. In this regard, shungite carbon in the form of ShC NPs [10] is of particular interest. 

The aim of this work is to show “in vitro” that ShC NPs can serve as a regulator of important biophysical processes when interacting with serum albumin as the main protein of blood plasma studied by DLS, Raman spectroscopy, EPR of spin labels and probes, and microcalorimetry. It is assumed that a wide range of functional specializations of albumin, from the transport and antioxidant protein to osmoregulatory and structure-forming protein [20,21], will make it possible, using this universal protein as an example, to reveal the main abilities of ShC NPs to manifest biological activity at the level of solvent-dependent molecular-colloidal mechanisms of interaction. The graphene molecules themselves, as the basic structures of ShC, may not have pronounced biological activity. However, as the organization in molecular colloidal systems becomes more complicated, the nanoparticles formed from them acquire some functional properties characteristic of macromolecular biopolymers and their supramolecular complexes.

The results obtained provide information that the incorporation of molecules into defects in the water hydrogen bond system allows this water system to control the incorporation processes either by changing macromolecular conformation or by the emergence of supramolecular or cluster organization in the newly created defects. The formation of a multilevel heterogeneous cluster system of ShC NPs is a thermodynamic process (although it is accompanied by kinetic phenomena). Most likely, this can be described using a phase diagram, which has much in common with the diagram for globular proteins [26,27,28] discussed in Section 2 for the case of serum albumin. A diagram of this type was used to explain the appearance of metastable clusters in fullerene dispersions [29] and in the dispersions of ShC NPs [10].

### 1.2. Structure-Function Properties of SA

**Figure 2:**
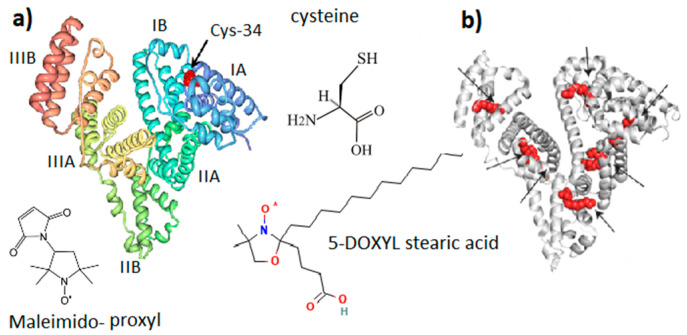
General molecular structure of serum albumin (SA). (**a**) SA domain structure with Cys-34 located in the IA domain cavity. (**b**) Potential binding sites of fatty acid in the SA structure. Maleimido-proxyl is a spin label that modifies the SH group of Cys-34. 5-DOXYL stearic acid is a spin probe adsorbed at fatty acid binding sites. Structures (**a**,**b**) are adapted from [30]. Different colors are provided for ease of observation.

Serum albumin (SA) is a transport protein in the blood. Its content is 55–60% of the total amount of proteins in plasma, and the concentration is about 30–50 mg/mL [20]. The ability of albumin to bind chemical compounds of various structures: hormones, metabolic products, toxins, drugs, metal ions, and fatty acids (FAs), as well as to maintain a sufficiently high osmotic pressure in plasma, comparable to the pressure in the cytoplasm, determines the variety of albumin functions in the vital activity of organisms [20]. The serum albumin is a poly-functional communicative and integrative molecular system that performs a homeostatic function by regulating both inter-tissue and inter-organ processes, and the functional and metabolic activity of inter-tissue processes [30]. SA has repeatedly served as a model protein for studying the osmotic properties of biopolymer solutions and the mechanisms that cause the behavior of protein solutions to deviate from ideal [31,32,33]. Based on its physicochemical parameters, various models have been constructed for the theoretical and experimental analysis of protein–protein intermolecular interactions [34]. Thus, SA solution is a completely self-sufficient system for studying the molecular mechanisms of homeostatic reactions in the presence of metabolites, drags, and nanoparticles of various natures. Basic details of the molecular structure of albumin relevant to this study are shown in Figure 2.

Water albumin solutions were found to contain significant fractions of dimers and high-order oligomers. Human SA is the most capable of oligomerization. In addition, the protein tends to form thixotropic structures, the structural elements of which are protein associates—clusters [35,36]. Cluster formation is probably a thermodynamic process of phase transition described by phase diagrams and relevant to protein conformation [26,27,28]. The structural heterogeneity of SA, caused by both genetic and postsynthetic factors, leaves many questions regarding the relationship between heterogeneity and functional properties, as well as the mechanism of “unloading” from ligands [37].

The albumin heterogeneity does not make it possible to obtain high-quality protein crystals suitable for a high-resolution X-ray diffraction analysis. Nevertheless, great efforts to obtain more than half a dozen different crystalline forms of SA contributed to the identification of the X-ray structure of SA with a resolution of 0.25 nm [38]. It was confirmed [39] that the three-dimensional structure of SA with dimensions of 14 nm in length and 4 nm in diameter is organized into three main domains (I, II, III), each containing two subdomains (A and B). Heart-shaped subdomains are built from 3–4 α-helices. The structures of the main domains coincide with mutual superpositions. Subdomains IA, IB, and IIA are densely grouped and form a “head”, while subdomains IIB, IIIA, and IIIB form an elongated “tail”.

Domain I of SA is believed to be responsible mainly for the binding of metal ions [40]. Domain II and III both have pockets formed by hydrophobic and positively charged residues in which different compounds, such as bilirubin, pyridoxal, and partially fatty acids, can be accommodated. Three tentative binding sites for FA, each with different surroundings, are located at the surface of each domain. In the presence of FAs, SA is an equilibrium mixture of macromolecules with different degrees of binding site occupation, which are in different conformations. The binding of FAs changes the charge equilibrium in the protein and, as a result, changes the interdomain interactions and the conformation of the protein [41]. As a result, different fractions of the protein can appear. 

Along with maintaining colloid–osmotic tissue homeostasis and binding and transporting physiologically important molecules and ions, SA has an important antioxidant function due to the ability of the SH group of the amino acid cysteine (Figure 2) to interact with free radicals and reactive oxygen species [30,42]. Human SA (HSA) is a single polypeptide chain with 585 residues containing 35 cysteines, which form 17 disulfide bridges, leaving the SH group of cysteine in the 34 position of the polypeptide chain (Cys-34, Figure 2) free. It is localized in a hydrophobic cavity about 1 nm deep in domain I [43]. The binding of one and two fatty acids (5-DOXYL stearic acid in Figure 2) significantly affects both the opening of the Cys-34 cavity and the global unfolding of the albumin structure, which increases the mobility of the atomic groups that form the Cys-34 cavity. In terms of structure and function, HSA and bovine SA (BSA) are very similar [44]. BSA is formed from 583 amino acid residues, 76% of which are identical to HSA. On the other hand, a number of differences in the binding pockets, surface structures, and charge distribution were found. Since HSA contains one tryptophan, this protein is more convenient for optical fluorescence studies. However, for studying the interaction of a protein with other molecules and nanoparticles, BSA is preferable because it is less hydrophobic and less prone to aggregation and self-association, forming particles with smaller sizes and narrower polydispersity. Therefore, BSA was mainly used in our study.

The structural-dynamic state of albumin molecules is characterized by a number of reversible conformational transitions in the region of non-physiological pH [32,44]. At neutral pH, SA exists in the neutral (N) form. At basic pH, the N form shifts to the basic (B) form (at pH 8) and to the “aged” (A) form at pH 10. At low pH (4.3), SA undergoes a transition to the fast (F) form and the extended (E) form at pH 2.7. For both acidic and basic transitions, SA undergoes an unfolding and loosening of its structure (see Figure 2 presented in ref. [32]). 

Increasing the temperature to 55 °C causes a gradual and reversible change in the protein secondary structure. The most significant change includes more content of helix secondary structure (about 3%) at 40 °C relative to that at 45°. It is accompanied by the reversible separating of I and II domains around 42–43 °C [45,46]. The region of 58–65 °C is characterized by a sharp onset of irreversible denaturation processes accompanied by protein aggregation [47]. 

FA binding under physiological conditions also causes local and global changes, manifested both at the level of the relative mutual displacement of I, II, and III domains of the SA structure and in smaller-scale changes in the region of the SH group of the Cys-34 [42]. This SH group is believed to be in two microstates: the reduced form, predominantly in the closed cavity, and the oxidized form when the cavity opens. The nearby amino acid residues histidine (His-39) and tyrosine (Tyr-84) located in the IA domain also affect the reactivity of the SH- group of Cys-34 [42]. Interactions with FAs not in direct contact with Cys-34 also induce conformational changes in its environment that promote the formation of -S-S-disulfide [48]. Albumin oxidation at other amino acid residues also affects, to some extent, protein thermal stability, resistance to aggregation, and aggregate morphology [49].

## 2. Results and Discussion

### 2.1. Effect of ShC Nanoparticles on Fatty Acid Binding by Albumin

**Figure 3:**
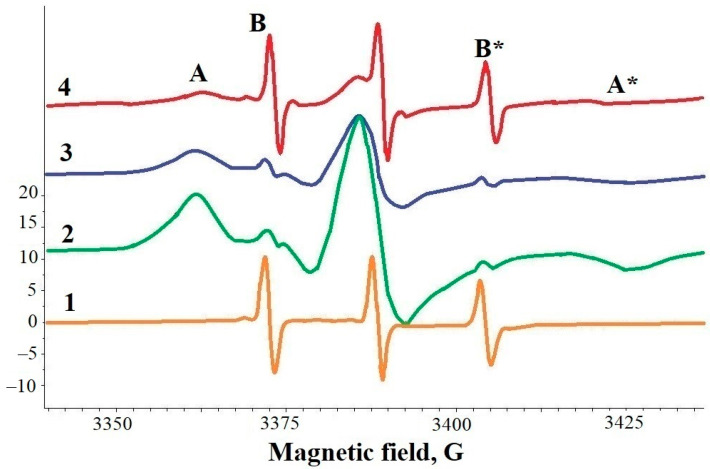
EPR spectra of 5-doxyl-stearic acid (5DSA) spin-probe in different samples: 1—dispersion of shungite carbon nanoparticles (ShC NPs); 2—dispersion of bovine serum albumin (BSA) with molar ratio 5DSA/BSA ≈ 2; 3—dispersion of BSA with molar ratio 5DSA/BSA ≈ 1; 4—dispersion of BSA + ShC NPs. A, A* and B, B* are spectral components (lines), wide and narrow, respectively, due to the immobilization of 5DSA in specific binding sites (A, A*), the relocation of 5DSA between binding sites (B, B* in spectra 2,3), fast rotation of spin probes in ShC NPs.

For the first time, the interaction of FA with ShC NPs was discovered by the EPR spin probe method [50,51], where the paramagnetic molecule 5-DOXIL stearic acid (5DSA) was used as the reporter group. Typically, the spin probe 5DSA, initially dissolved in chloroform, precipitates on a glass surface as a film after evaporation of the solvent. If a drop of dispersion of ShC NPs was applied to the film with 5DSA, then a part of the 5DSA from the film was adsorbed on the ShC [50,51]. As a result, three narrow equidistant spectral lines of the EPR spectrum of 5DSA with different amplitudes were observed in the drop of the ShC dispersion. This indicates the fast mobility of the probe (spectrum 1 in Figure 3). The spectrum turned out to be similar in appearance to the spectra of the 5DSA probe in the isotropic liquid crystal phase [52]. In the absence of ShC NPs, the spin probe did not pass from the film into a drop of bulk water, and a spectrum similar to spectrum 1 in Figure 3 was not observed. 

If a drop of bovine SA (BSA) dispersion was applied to a film with 5DSA, spin probes were adsorbed from the film by the protein. In this case, wide A-components appear in the EPR spectrum, indicating that the spin probe is localized in binding sites (spectra 2 and 3 in Figure 3). The presence of B-components, albeit weakly manifested, indicates that 5DSA migrates between binding sites [53]. Spectrum 4 in Figure 3 is the EPR spectrum of 5DSA in a mixed dispersion of BSA and ShC. It turned out that ShC NPs and BSA are able to compete for the adsorption of 5DSA. With an excess of BSA in the dispersion, 5DSA is predominantly adsorbed on BSA, but with an increase in ShC NP concentration, the spin probe is redistributed towards its binding to the latter.

A detailed analysis of the electron-spin parameters of the radical fragment of the hydrophobic spin probe 5DSA and hydrophilic spin probe 4-oxo-TEMPO [50,51] shows the difference for the protein and ShC NP microenvironment in the vicinity of the spin probe location. A higher homogeneity of the microenvironment and increased mobility of the probe in the protein–nanoparticle mixture are noted, apparently associated with the shielding of the probe from contacting water as part of the protein corona [51] in Figure 1.

**Figure 4:**
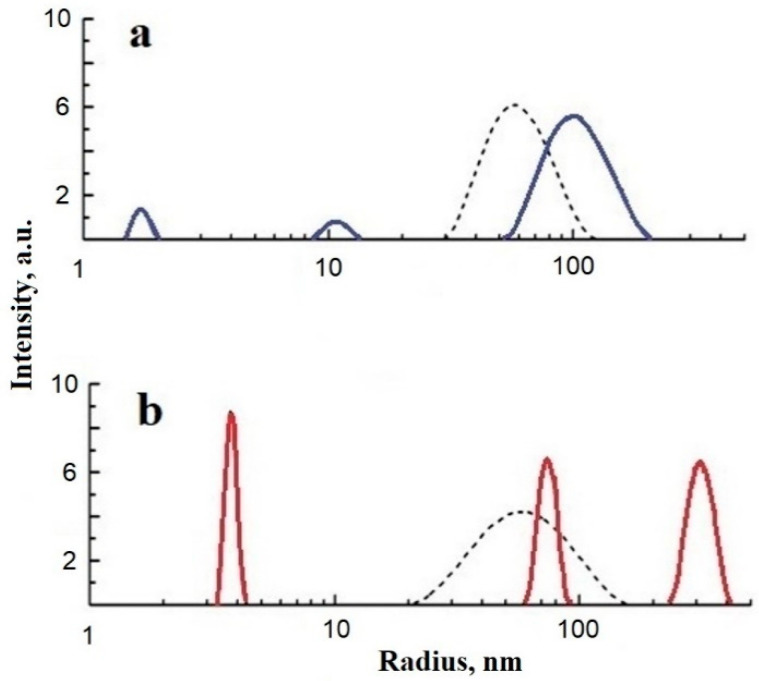
Relative intensity of scattered light vs. particle size (stokes radius) for bovine serum albumin (BSA) (**a**) fatty acid-free BSA (blue solid lines) and (**b**) fatty acid-containing BSA (red solid lines). Dashed lines present the distributions after shungite carbon nanoparticle (ShC NP) administration to the BSA dispersion. Concentration: BSA 1 g/L, ShC NPs 0.06 g/L (25 °C, pH 6).

DLS data of dispersions of fatty acid-free albumin and fatty acid-containing albumin show (Figure 4) that three types of protein particles of different sizes, including protein molecules and their larger associates, form one type of medium-sized associate under the effect of ShC NPs, although with a wider distribution. It follows that in the presence of nanoparticles, the distribution of protein associates over their size becomes much more homogeneous.

Thus, a mixed dispersion of SA and ShC NPs is more homogeneous than an SA solution separately. This is manifested both in the spread of the sizes of molecular associates and in the state of the microenvironment of FAs associated with proteins. The formation of the SA–ShC NP interface probably leads to greater homogeneity of FA binding sites and a decrease in protein fractions and “hot spots” on the protein surface, which are responsible for the supramolecular heterogeneity of the protein in solution. Although SA has about 7 FA binding sites, usually 1–2 sites are involved. Since, in organisms, there is an excess of albumin molecules compared to the possible concentration of nanoparticles, the effect of the latter in transport competition with SA as a whole can be considered insignificant. However, the importance of protein–nanoparticle interactions in the distribution of FAs may be manifested in the fact that, depending on the number of bound FAs, the conformation of proteins and their ability to form different fractions changes. Fractionation can be related to the phase properties of protein systems, causing the presence of liquid–liquid phase transitions, which will be discussed further. The latter is associated with the formation of membrane-less organelles in the cytoplasm.

### 2.2. Thermodynamic Evidence of ShC Effects

**Figure 5:**
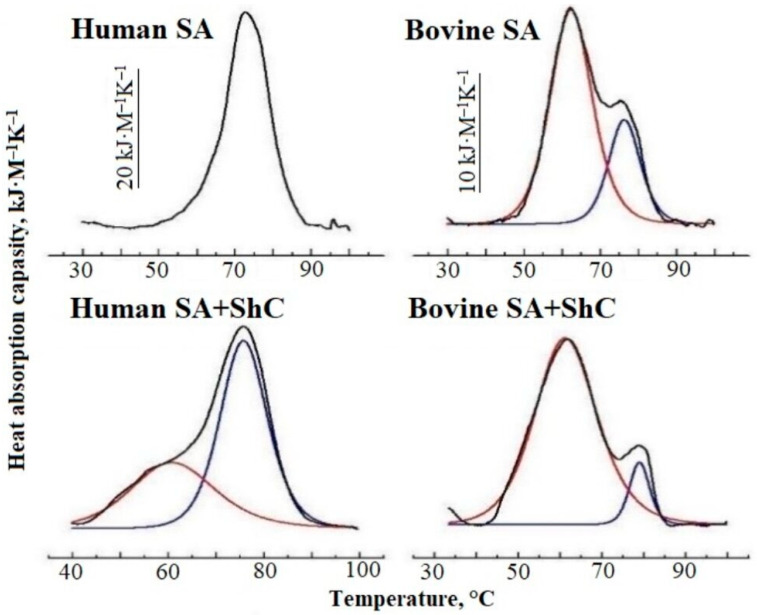
Heat absorption capacity of native Human and native bovine serum albumin (BSA) solutions (0.5 g/L, pH 6.5) in the absence and in the presence of shungite carbon nanoparticles (ShC NPs) (black curves). Red and blue curves are the Gaussian components.

Figure 5 shows the heat absorption curves of native solutions containing 1–2 FA molecules per molecule of human and bovine serum albumin [51]. It can be seen that in the presence of ShC NPs, the initially lonely HSA peak splits into two. Using the deconvolution procedure, two heat absorption peaks can be obtained. Such splitting of thermograms is well known for serum albumin from donor plasma and is due to the existence of two protein fractions containing different numbers of FAs per protein molecule. In this case, the low-temperature peak reflects the heat absorption of the fraction with a smaller number of FAs per molecule, and the high-temperature peak—with a larger number. The lower the FA saturation, the greater the intensity of the first peak. This suggests that in the presence of ShC NPs, the increase in the intensity of the first peak toward low temperatures may be due to the transition of part of the FA into the structure of nanoparticles with an increase in the protein fraction with a low FA content. A similar conclusion follows from the DSC data with BSA; however, the DSC spectrum of BSA is more difficult to interpret due to the overlap of melting peaks of individual structural domains. The obtained DSC data correlate with the data of the spin probe EPR method.

Thus, the study showed that ShC NPs affect the binding of FAs to SA, as well as the albumin conformation and aggregation caused by the transfer of part of the FAs from the protein to nanoparticles. Fatty acids are able to be transferred from protein to ShC NPs directly in the aqueous medium. 

### 2.3. Similarity of Redox Properties of ShC Nanoparticles and Serum Albumin

The antioxidant and cytotoxic activity of shungite extracts has been reported elsewhere [54]. For the first time, redox properties of ShC NPs were discovered by EPR spectroscopy for model systems of water–spin probe–ShC NPs when the radical oxidation/reduction of the nitroxide spin probe 5DSC was triggered by the introduction of divalent iron ions into the system [55]. Thus, the initial reduction of the NO* group of the spin probe to hydroxylamine with the loss of the EPR signal was again replaced by its oxidation to nitroxide with the appearance of an EPR signal upon the addition of a drop of cold water saturated with oxygen. In this case, the reaction rate increased significantly in the presence of ShC NPs both in the forward and reverse directions. Additionally, it was shown [56] that ShC NPs can significantly enhance the oxidation of blood proteins. This can occur due to both a direct increase in the degree of heme iron oxidation of hemoglobin and a decrease in the degree of free radical oxidation of the SH group of albumin Cys-34.

The antioxidant capacity of SA is known to be caused by the interaction of SH groups with free radicals and reactive oxygen species. The binding of FA has a significant effect on both the SA conformation and the oxidation of the SH group [57]. As for the SA reduction, the loss of paramagnetism of the nitroxyl radical of the spin probe with increasing temperature was found [58]. The latter depends on the conformation state of the microenvironment of the SH group and its accessibility to the solvent. Albumin oxidation, including that of other amino acids, affects the protein’s thermal stability, resistance to aggregation, and aggregate morphology [49]. At the same time, the interaction with FAs, which are not in direct contact with Cys-34, reveals the conformational changes in its environment as well, which contribute to the formation of disulfide [48]. 

The kinetics of a decrease in the integral intensity of the EPR spectrum of the spin probe 5DSA in complex with albumin at 62 °C were studied. It turned out that in the presence of the nanoparticles, the rate of decrease in the number of paramagnetic centers was significantly lower than in the absence of ShC NPs, thus revealing the effect of the latter in relation to Cys-34 as an oxidizing agent.

**Figure 6:**
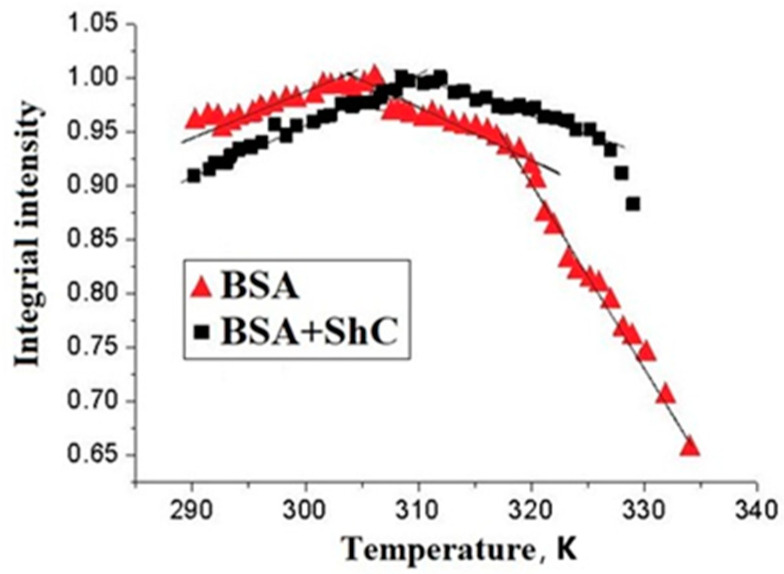
Temperature dependence of the integral intensity of EPR spectrum of a spin probe 5DSA bound to bovine serum albumin BSA) in the absence (triangles) and in the presence (squares) of shungite carbon nanoparticles (ShC NPs) in protein solution. Protein concentration is 50 mg/mL; 0.1 M NaCl; pH 6; probe/protein ≈ 1. The concentration of ShC NPs in the aqueous dispersion is 0.01 mg/mL Sections of the curves are approximated by straight lines to show the deviation of the dependencies from linearity.

The fragmentation of the dependences in Figure 6 corresponds to thermally induced conformational transitions of BSA in the range of 35–47 °C (308–320K). This range is 7–10 degrees lower than the transitions (around 315 K (42 °C)) typically seen at lower protein concentrations. They are associated with the partial opening of the Cys-34 cavity [45,46]. The deviations are probably associated with a decrease in the number of spin probes in the paramagnetic state due to the reduction of the doxyl NO* group of the probe to the diamagnetic form upon interaction with the SH group of albumin Cys-34. Thus, the interaction of SH of Cys-34 with the nitroxide group of 5DSA may manifest itself when the Cys-34 cavity opens during the conformational transition. At the same time, the NO* group of the probe begins to be reduced to hydroxylamine either during its diffusion between the binding centers [59] or, more likely, during the interaction between 5DSA and SH of Cys-34 located on different protein molecules. The formation of a protein corona around ShC NPs can cause the shift of the conformational transition that occurs in the range of 5–7 K [60].

Thus, the data on changes in the paramagnetism of a spin probe 5DSA during the oxidation of the SH group of the Cys-34 amino acid residue of domain I indicate that ShC NPs are able to exert a noticeable effect on the conformational transitions of SA with a change in the degree of accessibility of the Cys-34 SH group. In this case, ShC NPs act as oxidizing agents with respect to SA and affect the degree of SH oxidation by the 5DSA spin probe. ShC NPs and spin probes 5DSA compete with each other during the oxidation of the SH group. The formation of a protein corona can be a factor in the oxidation of SA by affecting the temperature of its conformational changes. It follows that the physiologically significant result of the interaction of ShC NPs with SA may be not so much their effect on oxidative mechanisms but rather their influence on the phase properties of biodispersions associated with the appearance of membrane-less organelles or other possible supramolecular structures.

### 2.4. Phase Properties of the Dispersions under Study

#### 2.4.1. Liquid–Liquid Phase Separation in Protein Dispersions

In recent years, the existence of liquid–liquid phase separation (LLPS) in cellular systems leading to the formation of condensed liquid droplets (“membrane-less organelles”) containing protein, RNA, and other biomolecules in high concentrations (Cajal bodies, nucleoli, P-granules, etc.) has become generally recognized [61,62,63]. They are periodically formed in the cytoplasm and exist without a separating membrane. This is a special form of compartmentalization in the cell, which contributes to the implementation of its various functions. A special role of proteins with an intrinsically disordered structure in these processes is emphasized [64,65]. 

However, LLPS also occurs in the dispersions of ordinary globular proteins in the native (folded) state. There are examples of protein dispersions with both upper critical solution temperature (UCST) and lower critical solution temperature (LCST), depending on their solubility. Solubility of some globular proteins (sickle cell hemoglobin [66], α-elastin [67]) decreases with increasing temperature, and they condense at LCST. Another group of proteins (lysozyme, crystallins [68], immunoglobulins [69]) dissolves better with increasing temperature, and they condense at UCST. The amino acid sequence is supposed to determine the nature of temperature dependence of solubility and condensation [70]. As an example, elastin-like and resilin-like peptides exhibit LCST and UCST phase behaviors, respectively [71]. Recently, however, there has been progress in the design of intrinsically disordered proteins (IDP), possessing both LCST and UCST, i.e., capable of condensing both during heating and cooling.

In general, LLPS in protein dispersions is known to be sensitive to variations in pH, temperature, ionic strength, state of crowding, and so on [64]. On the other hand, there are experimental data [72] and theoretical concepts [73,74] indicating that both UCST and LCST dispersions can be formed by the same protein depending on the microenvironment, i.e., by the composition and pH values of the solution (protein charge), the concentration and type of electrolyte, osmolytes, etc. Therefore, depending on the conditions, protein dispersions with both LCST and UCST are possible. Serum albumin is an example of a UCST protein in the presence of polyethylene glycols or a number of inorganic salts in the temperature range close to 0 °C [72]. On the other hand, the dispersion of SA shows LCST at higher temperatures in the presence of polyvalent salts [75]. It was assumed [28] that low-temperature and high-temperature folding intermediates can participate in phase transitions of the liquid–liquid type, leading to the formation of clusters from them in metastable equilibrium with the main phase of native proteins. 

#### 2.4.2. Solubility of Serum Albumin in Water–Salt Solutions 

**Figure 7:**
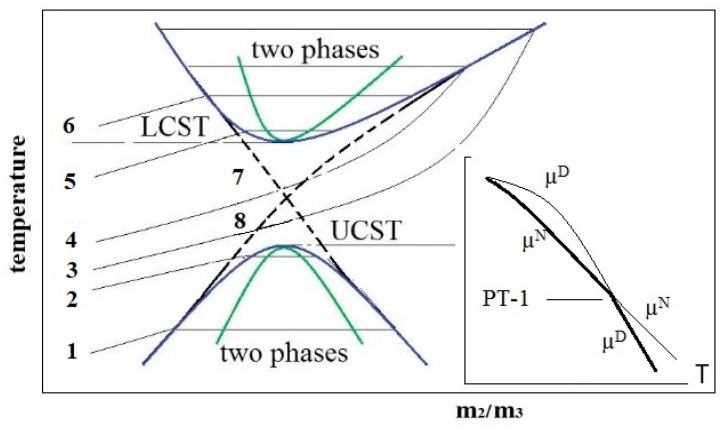
Hypothetical solubility phase diagram of the protein dispersion in the plane temperature—composition m_2_/m_3_, where m_2_ and m_3_ are the protein and salt molar concentrations, respectively. Binodals (blue parabolas) and spinodals (green parabolas) are represented as curves with upper critical solution temperature (UCST) and lower critical solution temperature (LCST). Curves 1–6 characterize the change in protein solubility with increasing (from bottom to top) salt concentration m_3_. They reflect the overall effect of “salting out”, “salting in”, and “salting out” as re-entrant phase separations induced by salt [78]. Curve 7 is the solubility of protein oligomers; curve 8 is the solubility of microfibrils. Insert: chemical potentials µ of native (N) and denatured (D) proteins on temperature.

A qualitative phase diagram (PD) of albumin solubility in water–salt solution in temperature–composition coordinates was proposed [28], characterized by the presence of spinodals, binodals, and two critical points, which present UCST and LCST modes (Figure 7). Diagrams of this type are typical for protein solutions [26,27] and are due to the existence of a long-range repulsive potential and a short-range attraction potential between macromolecules or particles known in the Derjaguin, Landau, Verwey, and Overbeek (DLVO) colloidal theory. The interaction of protein molecules in solution gives rise to their metastable associative clusters, possibly of micron size [76]. They are in metastable equilibrium with the main phase, which is shown in the PD in Figure 7 by horizontal segments connecting opposite sides of the binodal. In the spinodalregion, the two-phase stability is violated, and spinodal decomposition occurs with the formation of condensates. At temperatures below LCST and above UCST, a macroscopically single-phase system of protein molecules exists, although large-scale fluctuations in the concentration of molecules are possible in it [77].

Using standard physicochemical SA characteristics, the critical composition condition for the water–protein–salt system was theoretically obtained, which should correspond to the critical temperature in the phase diagram [79,80]. The ratio of molar concentrations of protein and salt, m_2_/m_3_, was established to be the control parameter of the LLPS. It depends on the charge of the protein, the number of electrolyte ions sorbed by the protein, and the salt activity coefficient. Later, in a number of experimental works [81,82,83], m_2_/m_3_ was shown to be the control parameter of the critical phase transition.

In [28], a thermodynamic approach to describing the PD in Figure 7 was presented in which the phase transition (PT) of liquid–liquid (L–L) type with UCST is a segregation-type PT and is due to the proximity of the first order PT between the native and denatured states of the protein. As shown in Figure 7 (insert), the chemical potentials of molecules in the denatured (D) and native (N) states change in such a way, thus intersecting at PT. At low temperatures, the D-state is stable, and at high temperatures, the N-state is stable. It is known that the first-order PTs do not end at a point since metastable states are possible [84]. In this case, protein clusters are associates containing protein molecules whose structure has not undergone a complete transformation into a more stable conformer under the given conditions but has remained in the molten or pre-molten protein globule state [85]. It is thermodynamically advantageous for such molecules to group into clusters, which can be in metastable equilibrium with the main phase [86], whose molecules have completely passed through the PT.

#### 2.4.3. Water–Albumin Interaction Revealed by Raman Scattering: The Role of ShC NPs

A study of the colloidal stability of albumin dispersions using Raman scattering in the wavenumber range 3000–3600 cm^−1^ was carried out in [87,88]. Analysis of spectral lines in this range, caused by OH stretching vibrations in the network of hydrogen bonds of water, showed the important role of the network in the supramolecular organization (formation of dynamic clusters) of protein dispersion. It is believed that the distribution of non-electrolytes during dissolution occurs according to the system of defects in the network of hydrogen bonds of water [89]. As defects are filled, the system of hydrogen bonds is first stabilized by optimizing the number of donor–acceptor bonds, but solubility stops in this case due to a decrease in entropy. If new, larger defects are created, solubility continues, but a colloidal system containing associates is already formed. Thus, it was found that the distribution of defects in the network of hydrogen bonds in water changes significantly non-linearly with protein concentration (see Figure 2 presented in ref. [88]). In the concentration range of 0.1–0.3 mg/mL of protein, the defects are filled, and the system of hydrogen bonds is stabilized to the greatest extent. The destabilization of the hydrogen bond system with further increasing protein concentration is attributed to its association [87,88], the degree of which was estimated from the data of the DLS method.

The interaction of the protein molecules themselves was considered within the framework of the concept of the formation of metastable protein associates (clusters) in newly formed defects in the network of water hydrogen bonds and associated phase separation of the liquid–liquid type (LLPS). In this case, protein clusters can be formations of the intermediate (or latent) phase, the sizes of which are smaller than the critical size necessary for the formation of a new phase nucleus in the classical representation of nucleation [90]. Such formations are characteristic not only of objects in the world of biopolymers and nanoparticles [91,92] but also of the mineral world [93]. In such seemingly completely different systems as dispersions of ShC NPs and SA, a generally similar behavior of the hydrogen bond network is observed with a change in the concentration of components. The primary associates that arise in this case have, on average, a close size and do not change with an increase in the concentration of the component. This may indicate that, first, primary defects in the hydrogen bond network are created by protein molecules or the basic structural units of ShC depending on their hydrophobic–hydrophilic balance and concentration, but then larger defects are formed that can accept larger associates. Colloidal stability to phase separation of protein solution is maintained by the formation of protein clusters of two main types and sizes as latent phase clusters (red and blue solid lines in Figure 4).

Nanoparticles, as a crowding element, can also induce phase transformations in protein solutions, which regulate the biochemical adaptation mechanism in biological media. Using the example of albumin and ShC NPs, we approached the problem of phase effects of protein and mixed (hybrid) dispersions from the point of view of the formation of nanobioconjugates and associates of the intermediate (hidden) phase. The colloidal stability of such clusters was assumed to be associated with the structural state of hydrogen bonds in water as well as with the ability of clusters to form defects in the hydrogen bond network. In the presence of NPs, the colloidal stability of the dispersion is mainly provided by the association of protein with NPs and the formation of a protein corona (Figure 4, single dashed lines). In this case, both stabilization and loosening of the network of hydrogen bonds of water occur more smoothly, without sharp extremes (see Figure 5 presented in [88]). Abiogenic NPs serve as centers of protein nucleation.

## 3. Materials and Methods

Bovine and human serum albumins (fraction V and essentially fatty acid-free lyophilized powders) were purchased from PAA Laboratories GmbH and Sigma-Aldrich Company, respectively, and used without further purification. Fraction V preparations of SA contain physiological concentration of FA of about 1:1 molar ratio to protein.

Hydrophobic spin-labeled 5-DOXIL stearic acid (DSA) purchased from Sigma-Aldrich was used for ESR spin probing of ShC nanoparticle interactions with BSA molecules in aqueous dispersion. The method for modifying BSA with DSA is described in detail in [51]. Deionized Millipore water (18.2 MΩ·cm resistivity) was used in all preparations of ShC nanoparticles and SA samples. Freshly prepared solutions of SA were used in all sets of experiments. All other chemicals and reagents were of analytical grade.

Aqueous ShC dispersions, applied for the discussed biomedical investigations, were prepared from carbon-rich shungite rocks following the original procedure described elsewhere [9]. Those were produced without additional chemical reagents and surfactants under water treatment due to the destruction of condensation contacts at different levels of the structural organization of shungite carbon [10].

**Figure 8:**
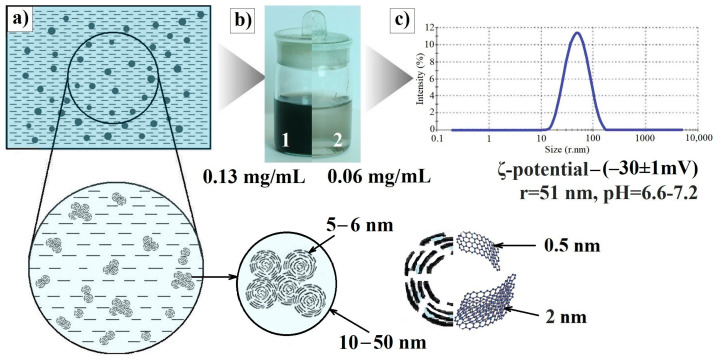
(**a**) Scheme of a diluted shungite carbon nanoparticle (ShC NP) dispersion. Characteristic sizes of NPs, globules, BSU, and stacks composing globules are below. (**b**) Aqueous ShC dispersions of initial—0.13 mg/mL (1) and diluted—0.06 mg/mL (2) concentration. (**c**) Particle size distributions in terms of the relative scattered intensity vs. size (stokes radius) for ShC NPs (DLS) and some characteristics of the dispersion used (pH, ζ-potential).

According to dynamic light scattering (DLS) data, NP mean radius constitutes ~51 nm with a polydispersity index of 0.25. Sedimentation properties (particle size distribution pattern, average particle size, pH value, ζ-potential) of the dispersion are quite stable over time (Figure 8).

The EPR spectra were recorded on an EMX 6/1 ESR spectrometer (Bruker BioSpin, Germany) with the temperature stabilization accuracy of the control system ±0.2 °C. We applied a modulation amplitude of 1 G and microwave power of 12.6 mW to avoid signal saturation and distortion. Preparation of the modified ShC dispersions is described in “Results and Discussion”.

The dynamic light scattering measurements were conducted using a particle size and zeta potential analyzer, Zetasizer Nano ZS (Malvern Instruments, Malvern, UK), equipped with a 633 nm He–Ne laser at 173° scattering angle. The DLS data were processed using the standard "Protein analysis" procedure of the Malvern Zetasizer software 6.01.

Raman spectra were recorded using a Nicolet Almega XR Raman spectrometer (Thermo Scientific, Madison, WI, USA), equipped with a 532 nm laser with a spectral resolution above 1 cm^−1^. The power of the exciting laser at this wavelength was 15 mW. The spectra were recorded with a 30min accumulation. The dispersions were placed in a quartz cuvette, which was installed in the compartment for macrosamples perpendicular to the laser beam axis. The temperature of the sample was 22 °C and did not change over laser. The Raman spectrum was obtained by excitation of the sample (dispersion of nanoparticles) at 532 nm, followed by measurements of the frequency and intensity of the scattered light. Changes in the position, intensity, and width of the Raman peaks in the spectral region 3000–3600 cm^−1^, which characterize the state of the water hydrogen bond system, were evaluated using OMNIC software (8.2).

The thermal denaturation of SA in water solutions and in ShC nanodispersions was studied with a concentration of 0.5 mg/mL.The sample preparation of the SA–ShC dispersions was performed in 2 mL tubes (Eppendorf, Hamburg, Germany) containing 2 mL of 0.1 mg/mL ShC nanodispersion and 1 mg SA. We used a NanoDSC (TA-Instruments, TX- Waters LLS 890W 410N Lindon, UT 84042 USA) differential scanning calorimeter equipped with 0.33 mL capillary platinum cells to record the microcalorimetry thermograms of aqueous SA solutions and mixed ShC dispersions. The heating rate of 1.0 K/min, temperature range of 20 °C to 100 °C, and constant pressure of 2 atm were used in all the scanning runs. No signs of endothermic or exothermic transitions were observed over the temperature range studied for ShC dispersions. Rescanning of the samples containing SA showed no reversibility of the protein endothermic transitions after cooling following the first scan. This re-run was used as baseline. Deconvolution of SA denaturation curves was carried out to analyze the characteristics of individual endothermic transitions of the protein. The enthalpy of constituting transitions and denaturation temperatures were calculated using the software package for microcalorimetric data processing, Nano Analyze TM Software version 1.0.11. The analysis was based on Gaussian functions.

The research was carried out using the equipment of the Core Facility, Karelian Research Centre RAS.

## 4. Conclusions

Thus, the interaction of soluble albumin with ShC NPs gives rise to the modulation of a number of potentially significant physiological effects: redox, conformational, ligand transport, and phase. It should be noted that these effects are interdependent: the change in protein conformation is associated with both the transfer of fatty acids and the antioxidant properties of the SH groups of albumin. Protein exchange between the corona of ShC NPs and solution causes a pH-dependent reversible transfer of fatty acids between BSA and nanoparticles, which is also facilitated by partial unfolding of the BSA conformation with increasing temperature. The ratio of protein and ShC NP concentrations controls the interaction with ligands as well as the degree of adsorption of these protein fractions on NPs. In real bio-liquids, the concentration of proteins is orders of magnitude higher than the possible concentration of nanoparticles, so the possible role of nanoparticles as modulators of transport or oxidative function is insignificant. It is a different matter if the local concentration of nanoparticles is high, for example, when creating biocompatible coatings for prosthetics or wound dressings. On the other hand, a physiologically significant result of the impact of ShC NPs on protein systems may be the influence on the phase properties of bio-liquids associated with the appearance of membrane-less organelles or other possible supramolecular structures. As it turns out, the transport, oxidative, conformational, and phase properties of protein systems are mutually related, but the last of these remains the least studied. One of the ways to solve this problem seems to be research in the direction of the properties of hydrogen bonds of water and their ability to arrange dissolved molecules in such a way that a certain energy optimum is achieved. Protein conformation affects the state of the water hydrogen bond network and the presence of defects in it, and thus the degree of formation of nanobioconjugates and associates that fill these defects. Their role as possible clusters of the intermediate (hidden) phase in the mechanism of colloidal stability of bionanodispersions and subsequent phase transitions, which provide the mechanism of phase regulation during biochemical adaptation, remains to be assessed. The results obtained show that ShC NPs have some similarities with BSA effects associated with transport, phase, colloidal, redox, and conformational properties. This can contribute to increasing the understanding of bionanohybrid systems, further interdisciplinary research in the field, as well as more targeted design processes involving ShC NPs in the development of bionanotechnologies and biomedical applications.

## Data Availability

No new data were created or analyzed in this study. Data sharing is not applicable to this article.
